# Deep Learning-Based Classification System for Facial Pigmented Lesions to Aid Laser Treatment Decisions

**DOI:** 10.7759/cureus.85428

**Published:** 2025-06-05

**Authors:** Haruyo Yamamoto, Chisa Nakashima, Kenichiro Kasai, Hiroyuki Irie, Hitonari Kanetomo, Shigeto Yanagihara, Sanae Miyake, Keiko Watanabe, Sayuri Sato, Hisashi Uhara, Fumiaki Takeda, Atsushi Otsuka

**Affiliations:** 1 Department of Dermatology, Kindai University Faculty of Medicine, Osaka, JPN; 2 Department of Plastic Surgery, Kasai Clinic for Plastic Surgery, Osaka, JPN; 3 Department of Dermatology, Graduate School of Medicine, Kyoto University, Kyoto, JPN; 4 Department of Dermatology, Kanetomo Dermatology Clinic, Osaka, JPN; 5 Department of Dermatology, Sapporo Medical University School of Medicine, Sapporo, JPN; 6 Department of Electronic Engineering and Computer Science, Faculty of Engineering, Kindai University, Higashihiroshima, JPN

**Keywords:** acquired dermal melanocytosis, convolutional neural networks, deep learning, densenet121, dermatological diagnosis, ephelides, facial pigmented lesions, inceptionresnetv2, melasma, solar lentigo

## Abstract

The accurate diagnosis of facial pigmented lesions is essential for selecting appropriate treatment strategies and improving patient outcomes. Deep learning algorithms, particularly convolutional neural networks (CNNs), have shown promise in classifying skin lesions but have been underutilized in differentiating facial pigmented lesions, particularly in the context of laser treatment planning.

The aim of this study is to develop and evaluate deep learning models using transfer learning to differentiate among five types of facial pigmented lesions and compare their diagnostic accuracy with that of expert and non-expert dermatologists.

A dataset comprising 432 high-resolution clinical images of five facial pigmented lesions, melasma, ephelides, acquired dermal melanocytosis (ADM), solar lentigo, and lentigo maligna/lentigo maligna melanoma (LM/LMM), was collected. Images underwent preprocessing, including white balance correction and region of interest (ROI) extraction. Two CNN architectures, InceptionResNetV2 and DenseNet121, were trained using transfer learning. The models' diagnostic accuracies were compared with those of nine board-certified dermatologists (experts) and 11 noncertified dermatologists (non-experts). The InceptionResNetV2 and DenseNet121 models achieved overall diagnostic accuracies of 87% and 86%, respectively. Both models outperformed expert dermatologists, who had a median diagnostic accuracy of 80%, and non-expert dermatologists, who had a median accuracy of 63%. Notably, both models achieved 100% sensitivity in identifying LM/LMM.

The developed deep learning models demonstrated superior diagnostic performance compared to dermatologists in differentiating among facial pigmented lesions. These findings suggest that such models have potential clinical applicability in assisting dermatological diagnosis and guiding appropriate treatment strategies.

## Introduction

Facial hyperpigmentation encompasses several common benign entities frequently encountered in clinical practice, including solar lentigo, melasma, ephelides, acquired dermal melanocytosis (ADM), and post-inflammatory hyperpigmentation. Accurate diagnosis is crucial as treatment strategies vary significantly depending on the lesion type. For example, while treatments such as pigment-specific lasers have shown promising outcomes for conditions such as solar lentigo and ephelides [1], inappropriate laser use can exacerbate melasma, and delaying necessary surgical excision for lentigo maligna/lentigo maligna melanoma (LM/LMM) due to misdiagnosis can have severe consequences [2,3]. Therefore, precise identification among these conditions is essential not only to differentiate benign from malignant lesions but also to select the most appropriate management strategy, avoiding potential complications and improving patient outcomes. Nonetheless, the visual similarity between these lesions often makes differential diagnosis challenging.

Recent advances in deep learning have significantly enhanced the classification and diagnosis of pigmented skin lesions, with algorithms achieving diagnostic precision comparable to that of dermatologists in identifying lesions suspicious for malignancy [4,5]. Convolutional neural networks (CNNs), in particular, have outperformed both general practitioners and dermatologists in skin lesion classification [6,7]. A pivotal study leveraged Google's Inception V3 (Mountain View, CA) and ImageNet transfer learning techniques to categorize 2,032 skin conditions, training the network with 129,450 images [8]. The resulting CNN model attained an average accuracy rate of 72.1% ± 0.9% across three categories of skin diseases, which was superior to the performance of two dermatologists who scored 65.78% ± 0.22% on the same validation subset [8]. These findings, along with those from related research, indicate that CNN architectures have the potential to equal or exceed the visual diagnostic accuracy of board-certified dermatologists in certain tasks [9].

While several studies have applied CNNs for skin lesion classification, including melanoma detection, relatively few have focused specifically on common benign and malignant facial pigmented lesions relevant to laser treatment planning [8,9]. Our study addresses this gap by including a diverse set of five facial lesions, melasma, ephelides, ADM, solar lentigo, and LM/LMM, and demonstrates superior performance using a limited dataset. This study introduces a deep learning model designed to assist in the diagnostic differentiation of facial pigmented lesions, with the aim of supporting clinical decision-making, particularly in contexts where laser therapy is being considered.

## Materials and methods

Patients and pigmented facial lesions

This study was approved by the Ethics Committee of Kindai University Hospital, Osaka, Japan (approval ID: R06-074). All methods were performed following the Ethical Guidelines for Medical and Health Research Involving Human Subjects; regarding data handling, we followed the Data Handling Guidelines for the Medical AI project. A retrospective analysis of clinical photographs was conducted from the date of ethics committee approval (September 6, 2024) to December 31, 2024. All clinical photographs were anonymized and used in a manner completely separated from personal information, ensuring that individuals could not be identified.

The clinical images were taken using standard digital cameras under routine clinical photography conditions. Efforts were made to ensure consistency, including using similar ambient room lighting and maintaining a relatively standard photographic distance and angle where feasible. Automatic camera settings were typically used. Images were stored as high-resolution digital images. Additionally, we confirmed that all images were of sufficient quality for accurate dermatological diagnosis by multiple experts. The target diseases are melasma, ephelides, ADM, solar lentigo, and lentigo maligna/lentigo maligna melanoma (LM/LMM). The breakdown of the extracted images was 82 melasma images (from 71 patients), 60 ephelides images (from 27 patients), 102 ADM images (from 102 patients), 104 solar lentigo images (from 55 patients), and 84 LM/LMM images (from 32 patients). This category included 10 cases of lentigo maligna (LM, in situ) and 22 cases of lentigo maligna melanoma (LMM, invasive). LM/LMM was biopsied and diagnosed histopathologically. The other pigmented lesions were diagnosed clinically and confirmed by five experts.

Training of a deep learning model

**Figure 1:**
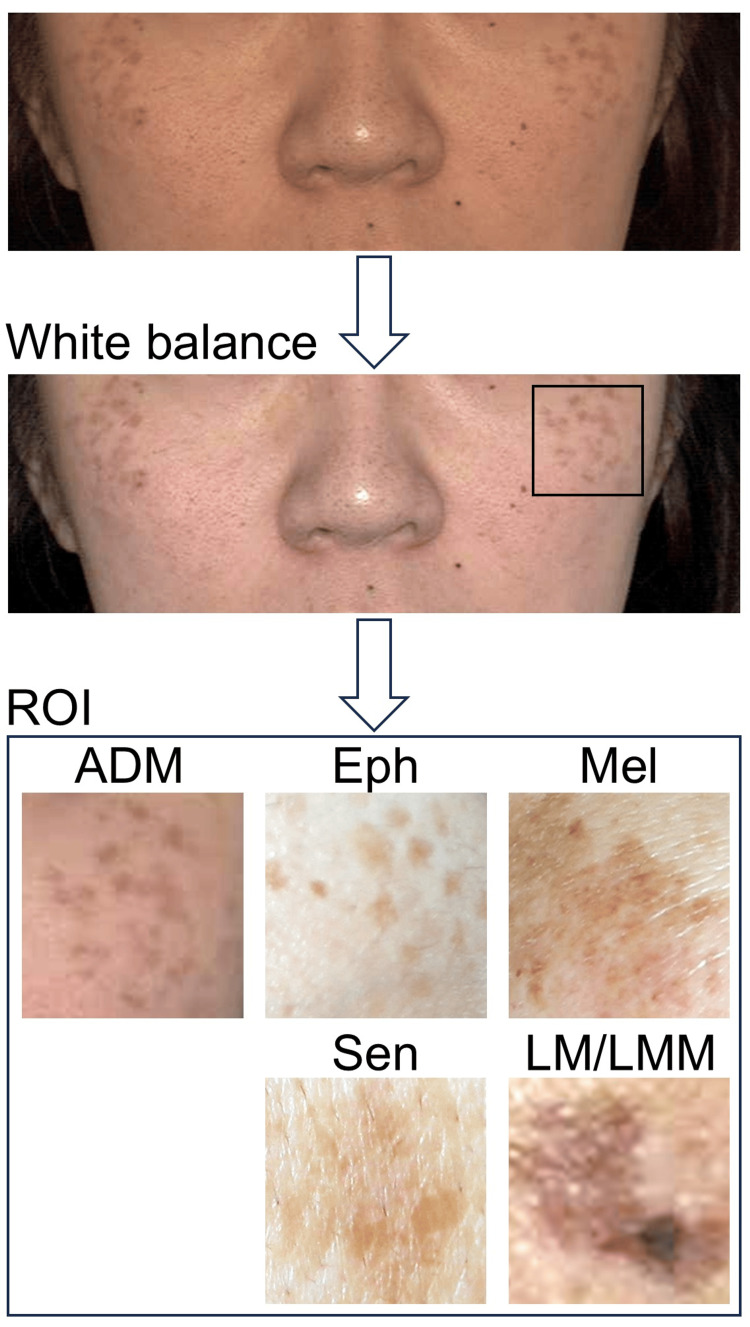
White balance correction and ROI extraction for CNN training Representative example of the image preprocessing workflow. The original facial image is corrected for white balance using Python's OpenCV. (Center) A region of interest (ROI), indicated by the square, is cropped to include the primary pigmented lesion. (Bottom) Examples of ROIs after white balance correction from each class of pigmented lesion: acquired dermal melanocytosis (ADM), ephelides (Eph), melasma (Mel), solar lentigo (Sen), and LM/LMM. Note that inherent variations in underlying skin tone and lesion pigmentation remain after correction. A square ROI was used to match the input requirements for the convolutional neural network (CNN). Subsequently, image blurriness was evaluated via Laplacian-based variance, and only sufficiently clear images (variance ≥ 10) proceeded to further analysis LM/LMM: lentigo maligna/lentigo maligna melanoma

All images were corrected for white balance using Python's (Python Software Foundation, Wilmington, DE) OpenCV (Intel, Santa Clara, CA) (Figure 1).

This preprocessing step aimed to normalize color representation across images potentially captured under slightly varying ambient lighting conditions, thereby reducing illumination-related variability. We cropped a region of interest (ROI) that included pigmented lesions from the entire image as a preprocessing step for training a CNN. A square ROI was used because a square image is generally applied as an input image to the CNN. The following processing was conducted on the designated ROI. Image blurriness was assessed using the image processing library, OpenCV. Each image was converted to grayscale, and the Laplacian filter was applied to enhance the edges of the image. Subsequently, image blurriness was evaluated objectively for all images using the variance of the Laplacian filter applied to the grayscale ROI. Only images meeting the quantitative clarity threshold (variance ≥ 10), including the examples shown, were utilized for further analysis.

For the processed images, transfer learning was conducted using either the InceptionResNetV2 or DenseNet121 models. This was achieved through Python scripting in Visual Studio Code (Microsoft Corp., Redmond, WA), utilizing TensorFlow and Keras. InceptionResNetV2 and DenseNet121, advanced CNNs with a multilayered and complex architecture, were employed as the base model. This model, pre-trained with weights from "ImageNet," did not include the top layer. Excluding the top layer allowed for adding a new output layer, tailored to a specific task: in this case, a five-class classification. The first 10 layers of InceptionResNetV2 or the first 100 layers of DenseNet121 were frozen (i.e., their weights were not updated during training), a common practice in transfer learning to leverage pre-trained general feature extraction capabilities, while the remaining layers were set to be trainable. Several dense layers and GlobalAveragePooling2D were added, each incorporating L2 regularization and dropout. The model was compiled using the Adam optimizer, with a learning rate set to 0.0001. A callback for learning rate scheduling, ReduceLROnPlateau, was configured. Custom preprocessing was conducted to process the image data, involving adjustments in hue, saturation, and brightness, and further enhance edge detection through the application of a Sobel filter. Subsequently, the ImageDataGenerator class was used for image data augmentation, which included rescaling (normalizing pixel values from 0 to 1), rotation, shift, shear, zoom, and horizontal flipping. The model underwent training with a batch size of 32 and for 50 epochs. The dataset images were randomly split into training (80%) and validation (20%) sets at the image level.

To further evaluate the performance of our models, we calculated the area under the receiver operating characteristic (ROC) curve (AUC) for each of the five classes. The ROC curves were generated by plotting the true positive rate against the false positive rate at various classification thresholds, and the resulting AUC values were used to quantitatively compare the diagnostic performance of InceptionResNetV2 to DenseNet121.

Diagnosis of facial pigmented lesions by dermatologists

The diagnosis of five types of facial pigmented lesions was performed by dermatologists. Twenty dermatologists participated in this study: nine were experts who held board certification as dermatology specialists by the Japanese Dermatological Association (expert group), whereas the remaining 11 were non-specialists (non-expert group). The expert group comprised seven dermatologists with 10-20 years of experience and two with more than 20 years of experience. The non-expert group included dermatologists with five or fewer years of experience. There was no time restriction for the dermatologists' answers.

## Results

Accuracy of the diagnosis of facial pigmented lesions in the artificial intelligence (AI) model InceptionResNetV2 and model DenseNet121

The accuracies of the AI models in the diagnosis of the five types of tumors (five-class discrimination) were 87% and 86% for the model InceptionResNetV2 and the model DenseNet121, respectively. The respective diagnostic accuracies of models InceptionResNetV2 and DenseNet121 in each type of facial pigmented lesions were as follows: 88% and 100% for the diagnosis of ADM, 82% and 73% for the diagnosis of ephelides, 100% and 100% for the diagnosis of LM/LMM, 80% and 80% for the diagnosis of melasma, and 75% and 80% for the diagnosis of solar lentigo (Table 1).

**Table 1:** Performance metrics (accuracy, sensitivity, specificity, and F1 score) for the classification of facial pigmented lesions by InceptionResNetV2 and DenseNet121 ADM, acquired dermal melanocytosis; LM/LMM, lentigo maligna/lentigo maligna melanoma

Lesion Type	Accuracy (%)	Sensitivity (%)	Specificity (%)	F1 Score	Model
ADM	88	86	95	0.85	InceptionResNetV2
	100	100	94	0.89	DenseNet121
		64	98		Expert
		53	96		Non-expert
Ephelides	82	82	100	0.9	InceptionResNetV2
	73	73	100	0.84	DenseNet121
		73	100		Expert
		58	98		Non-expert
LM/LMM	100	100	100	1	InceptionResNetV2
	100	100	98	0.97	DenseNet121
		83	98		Expert
		68	98		Non-expert
Melasma	80	80	86	0.67	InceptionResNetV2
	80	80	92	0.75	DenseNet121
		82	90		Expert
		62	89		Non-expert
Solar Lentigo	75	75	100	0.86	InceptionResNetV2
	80	80	100	0.89	DenseNet121
		93	88		Expert
		89	77		Non-expert

**Figure 2:**
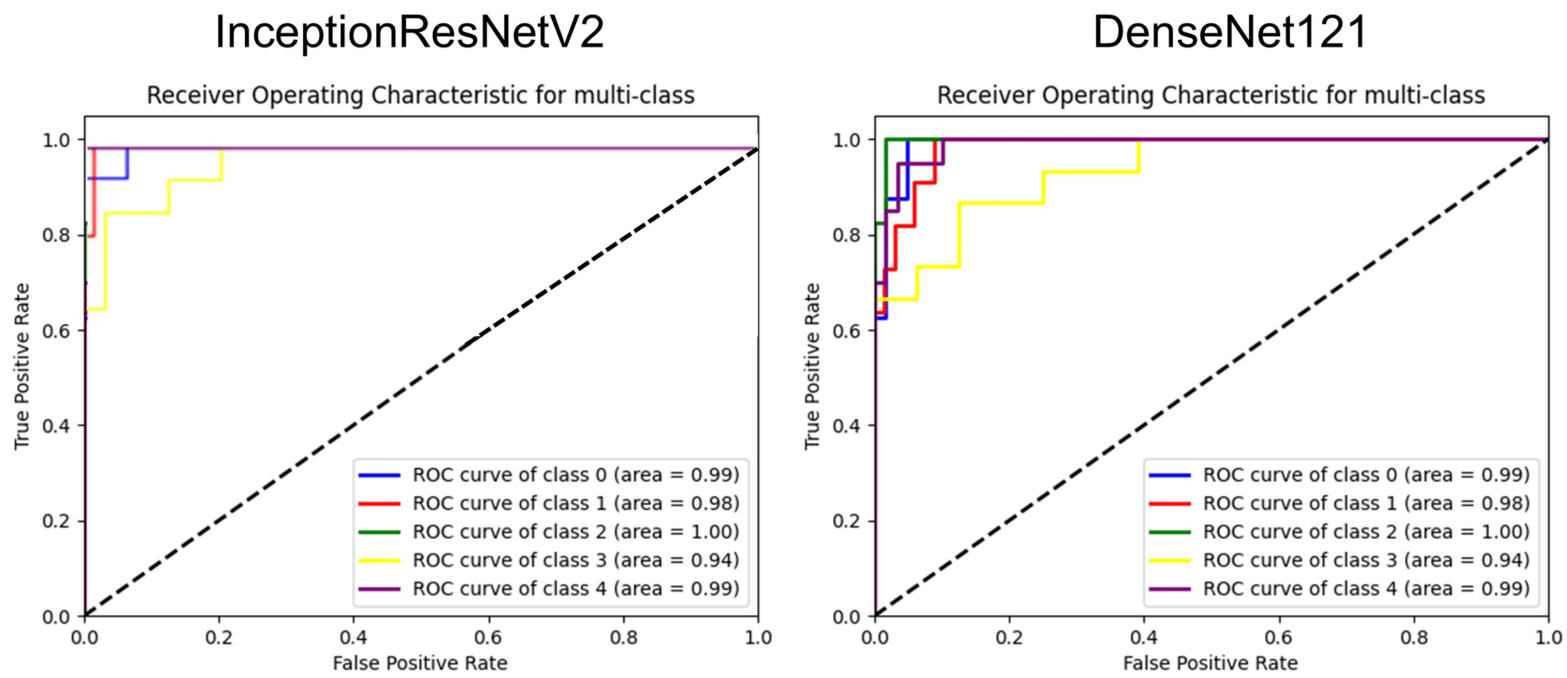
Performance comparison of InceptionResNetV2 and DenseNet121 models using ROC curve analysis for multi-class classification of facial pigmented lesions Receiver operating characteristic (ROC) curves for the multi-class classification of facial pigmented lesions (class 0, ADM; class 1, ephelides; class 2, LM/LMM; class 3, melasma; and class 4, solar lentigo). (Left) The InceptionResNetV2 model achieves AUC values of 1.00 (class 0), 1.00 (class 1), 1.00 (class 2), 0.97 (class 3), and 1.00 (class 4). (Right) The DenseNet121 model achieves AUC values of 0.99 (class 0), 0.98 (class 1), 1.00 (class 2), 0.94 (class 3), and 0.99 (class 4). The x-axis represents the false positive rate, and the y-axis represents the true positive rate. The diagonal dashed line indicates the reference line (AUC = 0.5) ADM, acquired dermal melanocytosis; LM/LMM, lentigo maligna/lentigo maligna melanoma; AUC, area under the receiver operating characteristic curve

The sensitivities for the diagnosis in each type of facial pigmented lesions were 88% and 100% for ADM, 82% and 73% for ephelides, 100% and 100% for LM/LMM, 80% and 80% for melasma, and 75% and 80% for solar lentigo by models InceptionResNetV2 and DenseNet121, respectively. Similarly, the specificities for each facial pigmented lesions are 95% and 94% for ADM, 100% and 100% for ephelides, 100% and 98% for LM/LMM, 86% and 92% for melasma, and 100% and 100% for solar lentigo by models InceptionResNetV2 and DenseNet121, respectively. The F1 scores for each facial pigmented lesions are 0.85 and 0.89 for ADM, 0.90 and 0.84 for ephelides, 1.00 and 0.97 for LM/LMM, 0.67 and 0.75 for melasma, and 0.86 and 0.89 for solar lentigo by models InceptionResNetV2 and DenseNet121, respectively. The ROC curves for both AI models are shown in Figure 2.

InceptionResNetV2 achieved AUC values of 1.00 for ADM, 1.00 for ephelides, 1.00 for LM/LMM, 0.97 for melasma, and 1.00 for solar lentigo. Similarly, DenseNet121 showed AUC values of 0.99 for ADM, 0.98 for ephelides, 1.00 for LM/LMM, 0.94 for melasma, and 0.99 for solar lentigo. These high AUC values in both models indicate robust discriminative ability across all five classes of facial pigmented lesions, although InceptionResNetV2 exhibited slightly higher AUC for ephelides and melasma compared to DenseNet121.

Comparison of the diagnostic accuracies of AI and dermatologists

**Figure 3:**
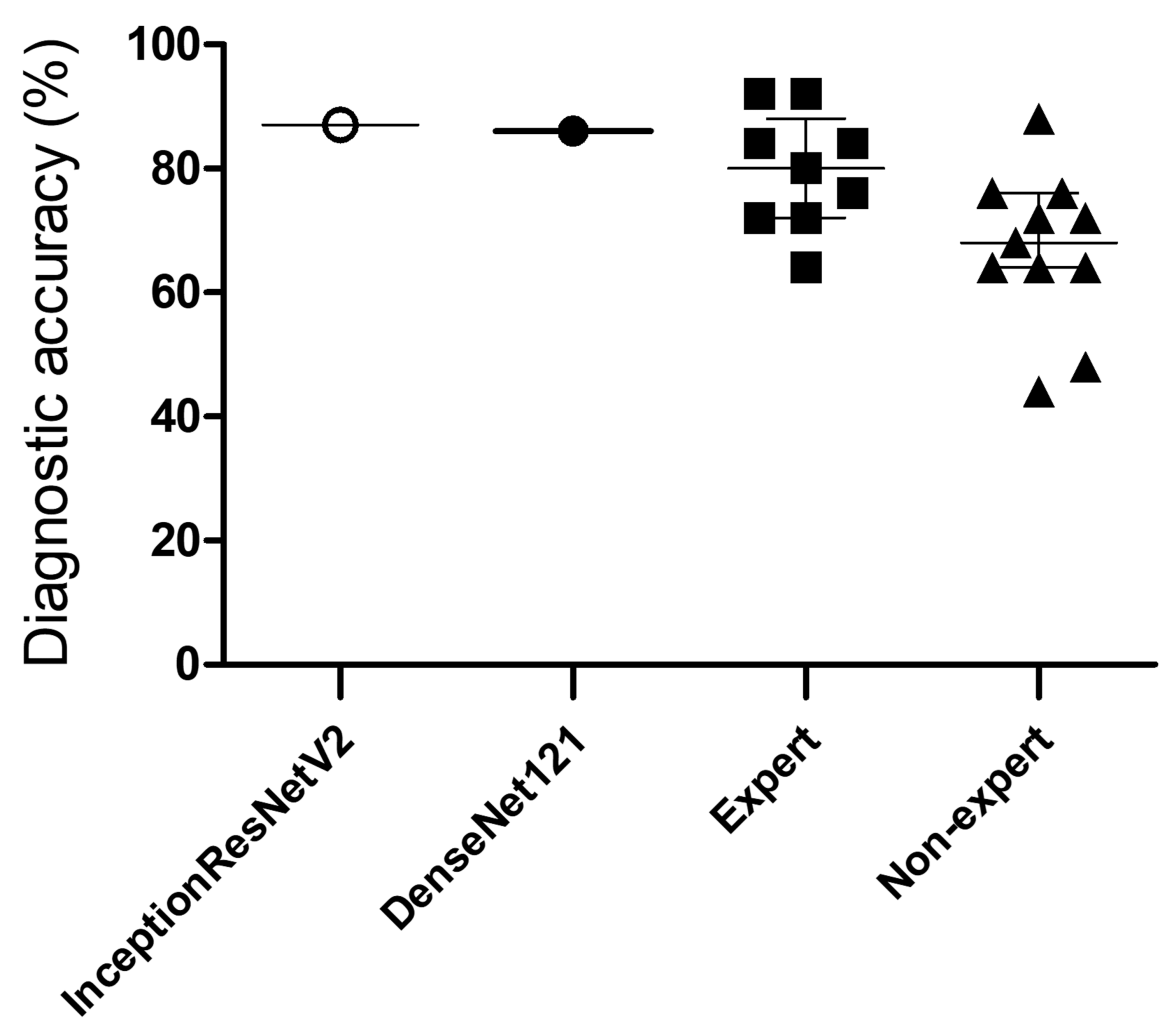
Comparison of AI models to dermatologists in the diagnosis of facial pigmented lesions Comparison of the diagnostic performances of two AI models (model InceptionResNetV2 and model DenseNet121) and 20 dermatologists for facial pigmented lesions. The overall diagnostic accuracies of the model InceptionResNetV2 and the model DenseNet121 were 87% and 86%, respectively. The median diagnostic accuracies of dermatologists were 80% for experts (certified by academic societies) and 63% for non-experts. The white circle and black circle represent each AI model, black squares represent expert dermatologists, and black triangles represent non-expert dermatologists AI: artificial intelligence

We compared the performances of the two AI models (model InceptionResNetV2 and model DenseNet121) and 20 dermatologists, which included nine experts certified by academic societies and 11 non-experts, for the diagnosis of facial pigmented lesions. The overall diagnostic accuracies of the model InceptionResNetV2 and the model DenseNet121 were 87% and 86%, respectively. The median percentages of the diagnostic accuracies of dermatologists were 80% and 63% for experts and non-experts, respectively (Figure 3).

A detailed analysis of performance metrics for each specific lesion type is presented in Table 1. This class-wise comparison revealed specific areas where the AI models demonstrated advantages. For example, while expert dermatologists achieved high specificity for most conditions (e.g., >97% for ADM, ephelides, and LMM), their sensitivity was notably lower than the AI models for ADM (64% versus 88% for InceptionResNetV2 and 100% for DenseNet121). Non-expert dermatologists generally exhibited lower sensitivity compared to both experts and the AI models across multiple categories, particularly for ADM (53%), melasma (62%), and ephelides (58%). Both AI models achieved perfect sensitivity (100%) for LM/LMM, surpassing both the expert (83%) and non-expert (68%) groups in detecting this malignancy. Conversely, expert dermatologists showed high sensitivity for solar lentigo (93%), comparable to or exceeding the AI models (75% and 80%).

## Discussion

In this study, we developed and evaluated a novel transfer learning system designed to differentiate between five types of facial pigmented lesions using a limited dataset. While numerous studies have applied deep learning to skin lesion classification and melanoma detection, including facial images, research specifically focused on the differential diagnosis of this common set of five facial pigmented lesions (melasma, ephelides, ADM, solar lentigo, and LM/LMM); using dedicated models remains relatively limited. Our results demonstrated that the system, employing InceptionResNetV2 and DenseNet121 models, achieved higher diagnostic accuracy than both dermatologists and dermatology residents. The diagnostic accuracies for the InceptionResNetV2 and DenseNet121 models were 87% and 86%, respectively, outperforming the median diagnostic accuracies of human experts, which were 80% for certified specialists and 63% for non-specialists. Notably, the models showed exceptional performance in identifying LM/LMM, achieving 100% sensitivity, highlighting their potential to assist in clinical settings where accurate diagnosis is critical for effective treatment planning. This indicates the potential applicability of our deep learning models in clinical settings to assist in the accurate diagnosis and treatment of facial pigmented lesions. Although our CNN model is not designed to select specific laser parameters or predict treatment outcomes, accurate diagnosis is a critical prerequisite for determining whether laser therapy is appropriate and selecting the suitable laser modality. Therefore, the system is intended as a diagnostic support tool, especially valuable in clinical contexts where laser treatment is being considered. The system is intended to assist not only board-certified dermatologists but also non-specialist clinicians in improving diagnostic accuracy for facial pigmented lesions, especially in settings where dermatological expertise is limited. It should be noted that our model is not designed to differentiate between laser types (e.g., Q-switched versus picosecond lasers).

Facial pigmented lesions encompass a variety of conditions, including melasma, ephelides, solar lentigo, ADM, and LM/LMM, each requiring distinct treatment approaches. It is particularly important to make an accurate diagnosis before proceeding with laser treatment for melasma. Melasma predominantly affects women in their 30s to 40s, appearing as symmetric brownish patches on the cheeks and around the eyes. It is often exacerbated by hormonal changes such as pregnancy or oral contraceptive use and can worsen with ultraviolet (UV) exposure or excessive skin care routines. Effective treatments include UV protection, topical bleaching agents such as hydroquinone, chemical peels, and oral medications such as vitamin C and tranexamic acid. However, laser or intense pulsed light (IPL) treatments can aggravate melasma, making accurate diagnosis critical before commencing treatment [10]. Both ephelides and solar lentigines are exacerbated by ultraviolet radiation exposure [11]. Ephelides present as small, symmetrically distributed brownish macules, predominantly appearing across the nasal bridge and malar regions. They occur more frequently in individuals with fair skin phenotypes and demonstrate seasonal variation, darkening with sun exposure and lightening during winter months. While melanogenic activity increases within these lesions, there is no corresponding increase in melanocyte density. Various treatment modalities, including Q-switched and picosecond lasers, as well as intense pulsed light (IPL) therapy, have demonstrated efficacy in reducing their appearance. Solar lentigines, commonly referred to as age spots, typically manifest on chronically sun-exposed areas such as the face, dorsal hands, and extensor forearms. These pigmented lesions develop as a consequence of cumulative ultraviolet exposure, resulting in DNA damage to both keratinocytes and melanocytes. Treatment approaches vary according to lesion characteristics: Q-switched and picosecond lasers are particularly effective for discrete, larger lesions, while IPL therapy is often the preferred modality for treating multiple lesions distributed across facial regions due to its broader coverage area and reduced downtime. In addition, ADM is characterized by brownish to grayish-brown spots symmetrically distributed on the forehead, eyelids, cheeks, and nose, predominantly in women aged 10-30. Unlike nevus of Ota, ADM does not affect the conjunctiva or oral mucosa. Treatment involves Q-switched lasers, which can penetrate the dermis where melanocytes reside.

Finally, lentigo maligna (LM), and its invasive form LM/LMM, must be carefully differentiated from these benign pigmented lesions as they represent in situ and invasive melanoma, respectively. LM typically presents as an irregularly pigmented macule or patch with varying shades of brown to black, often with asymmetric borders and geographic growth pattern, predominantly occurring on chronically sun-damaged skin of elderly patients. Key distinguishing features include progressive enlargement, irregular borders, color variegation, and asymmetry. While benign facial pigmented lesions can be safely treated with various laser modalities, LM/LMM requires surgical excision with appropriate margins as the definitive treatment. Dermoscopy and, when indicated, biopsy are essential tools for accurate diagnosis. Any facial pigmented lesion demonstrating atypical features and recent changes in size, shape, or color or failing to respond to conventional treatments should prompt immediate referral to a dermatologist for careful evaluation to rule out malignancy.

Despite the promising results, this study has several limitations. Firstly, and most significantly, the dataset was split at the image level, meaning images from the same patient could potentially exist in both training and validation sets. This represents a major limitation as it likely leads to an overestimation of the model's generalization performance on truly unseen patients. Future work must employ strict patient-level splitting. In addition, the dataset was relatively small, which may affect the generalizability of the findings. Although the deep learning models showed high accuracy, their performance may vary when applied to a larger, more diverse population. Secondly, the study was conducted using images captured under controlled conditions, which may not reflect real-world variations in image quality and lighting. In addition, while white balance correction was used to standardize color representation, the model's performance without this step or with variations inherent in real-world, uncontrolled image acquisition (where automatic correction might be inconsistent) was not assessed. Reliance on consistent preprocessing could affect performance in less controlled clinical environments. Thirdly, the exclusion of rare pigmented lesions and the focus on only five types may limit the applicability of the model to a broader spectrum of skin conditions. Additionally, while the training of these deep learning models demands significant computational resources, the inference (classification) stage used for diagnosis is substantially less intensive. Nonetheless, integration into clinical practice still necessitates adequate hardware and software infrastructure. Fourthly, our evaluation utilized a single train/validation split performed at the image level. Employing k-fold cross-validation in future work, critically combined with patient-level splitting as previously discussed, would provide a more robust and reliable assessment of the models' stability and true generalizability across different patient populations. Finally, while the models demonstrated superior diagnostic accuracy compared to dermatologists, the integration of these models into clinical practice necessitates further validation through prospective studies and real-world trials.

Furthermore, this study focused solely on diagnostic classification. Future research could explore the development of AI models capable of predicting the risk of potential adverse effects from specific treatments such as laser therapy (e.g., erythema, post-inflammatory hyper/hypopigmentation, and the Koebner phenomenon), which would complement the diagnostic capabilities and further aid in personalized treatment planning [12].

## Conclusions

In summary, the differential diagnosis of facial pigmented lesions is essential for appropriate treatment selection. The results suggest that our deep learning models hold substantial potential in supporting the accurate diagnosis of these lesions, potentially aiding dermatologists in making more informed treatment decisions and improving patient outcomes.
